# Comparing Sublingual and Inhaled Cannabis Therapies for Low Back Pain: An Observational Open-Label Study

**DOI:** 10.5041/RMMJ.10485

**Published:** 2022-10-27

**Authors:** Dror Robinson, Sivan Ritter, Mustafa Yassin

**Affiliations:** 1 Department of Orthopedics, Hasharon Hospital, Rabin Medical Center, Petah Tikva, Israel; 2 The Behavioral Neurobiology Laboratory, Department of Occupational Therapy, Faculty of Social Welfare and Health Sciences, The University of Haifa, Haifa, Israel

**Keywords:** Cannabis, CBD, low back pain, pain management, sciatica, THC

## Abstract

**Background and Objective:**

Medical cannabis is becoming an acceptable treatment modality in medicine, especially for pain relief. Concurrently, cannabis use is becoming more prevalent worldwide, a public demand-driven trend despite the lack of established scientific basis. This observational open-label study sought to investigate the effectiveness of cannabis therapy for alleviating low back pain symptoms.

**Methods:**

Two types of cannabis treatment modalities were sequentially administered to chronic low back pain patients. After an initial 1-month washout period (WO1), the first modality was cannabidiol (CBD)-rich sublingual extract treatment administered for 10 months. Following another washout period, the second modality, Δ^9^-tetrahydrocannabinol (THC)-rich smoked inflorescence (whole dried cannabis flowers) was administered for 12 months.

**Results:**

Enrolled in the study were 24 patients whose advanced imaging studies (i.e. computerized tomography or magnetic resonance imaging of the lumbar spine) revealed disc herniation or spinal stenosis. Three patients dropped out of extract therapy treatment but resumed study participation to receive THC-rich smoking therapy. After a minimum of 2 years, cannabis therapy had reduced lower back pain symptoms, as assessed by Oswestry Disability Index, the SF-12 patient-reported outcome questionnaire, and the visual analogue scale. Pain reduction was not significant during the extract treatment part of the study; however, pain reduction was significant during the inhaled therapy part of the study.

**Conclusions:**

Our findings indicate that inhaled THC-rich therapy is more effective than CBD-rich sublingual extract therapy for treating low back pain and that cannabis therapy is safe and effective for chronic low back pain.

## INTRODUCTION

Medical cannabis is rapidly becoming an acceptable treatment modality,^1^ including in Israel. This was demonstrated by recent reports from the Medical Cannabis Research Unit of the Israeli Ministry of Health (IMOH). They presented data showing that the number of medical cannabis licenses issued to patients between January and February 2022 had increased by 951, of which 705 (74%) were prescribed to treat chronic neuropathic non-cancer pain (CNNCP).^2^,^3^ Currently, active medical cannabis licenses have been issued to about 1.2% (110,971 patients) of the Israeli population, and 56.6% of such licenses were prescribed to treat CNNCP.^2^ Nonetheless, a major obstacle to the widespread legal use of medical cannabinoid-based (CB) therapy is the lack of sufficient evidence-based data.

### The Complex Multiplicity of Cannabinoids Research for Pain

Positive anecdotal outcomes reported by cannabis self-medicating individuals have propelled further studies of cannabinoid effect(s) on pain.^4^ However, the naturally occurring variation among and between the phytoconstituents of different cannabis cultivars^5^–^8^ makes it difficult to quantitate and compare studies and subjects^4^; this was demonstrated in a recent study of 429 CNNCP patients.^9^ The patients were treated with inhaled CB therapy for 6 months, which consisted of 41 cultivars in 350 cultivar combinations, of which 83% were Δ^9^-tetrahydrocannabinol (THC)-dominant and 17% were cannabidiol (CBD)-dominant. Despite unchanged pain intensities, there was a reported decrease in analgesic medication consumption and an increased quality of life, which coincided with higher doses of THC and the alpha-pinene terpene.^9^ Hence, this and other studies are needed to elucidate further specific cannabis therapy modalities, including their treatment outcomes after long-term treatment.

Additionally, the interactions among (intra-entourage effect) and between (inter-entourage effect) the phytoconstituents of cannabis cultivars (which also include terpenes, flavonoids, and hundreds of minor phytocannabinoids) may interfere with desired treatment outcomes, and therefore should be studied for each clinical indication.^6^ Phytoconstituents of cannabis, including phytocannabinoids, may inhibit some medications when co-administered. Inhibitory effects of major cannabinoids (such as THC, CBD, and cannabinol) on the enzymes involved in xenobiotic metabolism have been described, particularly regarding drugs that are substrates for cytochromes-P450-(CYP)2C19, CYP2C9, and CYP1A2 enzymes, as well as co-administration of medications metabolized by hepatic carboxylesterase-1 and/or by uridine-5′-diphospho-glucuronosyltransferase (UDP-UGT) enzymes.^10^ Nonetheless, it should be noted that CB treatments are potentially promising for reducing the chronic use of prescribed opioids for CNNCP.^11^,^12^ This was recently demonstrated in an observational study (*n*=3,544) that reported reduced medications use at six months, among which opioid use was reduced by 52.5%, analgesics by 39.2%, antipsychotics by 36.9%, antiepileptics by 35.7%, and hypnotics and sedatives were reduced by 35.3%.^13^

### Modalities of Cannabinoid-based Treatments

Cannabis can be administered in multiple ways. In Israel, inhaled cannabis is the dominant consumption modality. An alternative modality is extract administered sublingually. Oral consumption as edibles is reserved for children and was not relevant to the current study population.

Treating pain with sublingually administered cannabis extracts is preferred by many physicians, as it may be regarded as easier to obtain from the pharmacy, and to consume. Sublingual administration might also have the benefit of a more consistent dosing regimen while avoiding the adverse effects of smoking. The authors’ real-world clinical experience indicates a relative lack of efficiency of sublingual extract treatments compared to smoking. Most patients seem to prefer smoking cannabis to extract consumption for pain relief. This preference seems to be supported by recent data released by the Medical Cannabis Unit of the IMOH for January and February 2022. In reporting the total number of licenses issued for medical cannabis products, they noted that 89.5% and 89.7% of the licenses were for dried inflorescences in January and February, respectively, while only 10.3% and 10.1% were for extracts, respectively.^2^,^3^ This reflected a slight decrease in sublingual extract use and a slight increase in dried inflorescences. Interestingly, a recent survey of North American adults who self-administered cannabis for chronic pain (*n*=1,087) found that 58% of those surveyed used it to mitigate back pain, 36.1% used inhalation therapy, 45.1% used both inhalation and non-inhalation therapy, and 18.8% used non-inhalation therapy only.^14^ The cannabis usage differences between Israel and North America may be attributed to the greater variety of legally available CB products in the surveyed North American locations, such as edibles and high-concentration THC, which are not permitted in Israel. Despite the widespread use of inhaled (smoked or vaporized) CB therapy (by self-medication or by prescription), the relative efficacy of these two cannabinoid-consumption modalities remains unclear.

Currently, clinical trials data that compare administration methods are sparse. The current study was performed to assess the effect(s) of sublingual cannabis extract on low-back pain (LBP) as opposed to inhalation by smoking.

## METHODS

### Setting

This observational open-label study was conducted from 2017 to 2019 at the orthopedic clinic of Rabin Medical Center Golda Hasharon Campus, Petah Tikva, Israel. This study was conducted in full compliance and following the ICH-GCP standards and the ISO14155 Declaration of Helsinki ethical standard requirements (approval 17–20 RMC).

### Patient Enrollment and Inclusion and Exclusion Criteria

Patients considered eligible for the study signed a consent form to receive cannabis extract therapy (high CBD-to-THC ratio) and possibly, if clinically indicated, to receive dried inflorescence (high THC-to-CBD ratio) in the second phase of the study.

Inclusion and exclusion criteria for the study were aligned with the IMOH regulations and requirements for receiving CB therapy (Table 1). All patients underwent advanced imaging studies per the inclusion criteria to confirm pain-related anatomical abnormalities. None of the patients had received CB therapy prior to study enrollment.

**Table 1:** Study Inclusion and Exclusion Criteria Based on Israeli Ministry of Health Regulations.

Inclusion Criteria	
1.	>18 years of age
2.	Mild to severe LBP and/or sciatica
3.	Imaging studies (CT or MRI) that support symptomatic cause due to anatomical abnormality
4.	Unresponsive to standard therapy at least 1 month
5.	Physically and mentally willing and able to comply with treatment regimen and understand informed consent and study procedures
6.	Signed and dated informed consent form
**Exclusion Criteria**	
1.	Screening VAS pain score <6 (scale: 0=no pain; 10=worst imaginable pain)
2.	Known allergy to cannabis or its components
3.	Pregnancy or plans to become pregnant during the study
4.	Participant is breast-feeding or planning to breast-feed during the study
5.	Participant suffers from mental disorder that precludes administration of study drug
6.	Prisoners and soldiers due to free-will informed consent issues
7.	Inability to sign informed consent form
8.	Unstable angina pectoris, or cardiac insufficiency precluding cannabis administration, or congestive heart failure
9.	Immunosuppressed patients unless cannabis administration was deemed safe by the treating physician
10.	Known *Aspergillus* infection
11.	Panic attacks or anxiety, unless a psychiatrist authorized cannabis therapy following intake interview
12.	Any mental/psychiatric illness in first-degree relative of a young patient (<30 years old)
13.	Severe COPD as defined according to the Global Initiative for Chronic Obstructive Lung Disease (GOLD) stage 3, i.e. FEV-1 between 30% and 49%^15^
14.	Taking any of the following medications and/or natural remedies: primidone, phenobarbital, carbamazepine, rifampicin, rifabutin, troglitazone, and *Hypericum perforatum*

COPD, chronic obstructive lung disease; CT, computed tomography; FEV, forced expiratory volume; LBP, lower back pain; MRI, magnetic resonance imaging; VAS, visual analogue scale.

### Cannabis Therapy Schedule

**Figure 1:**
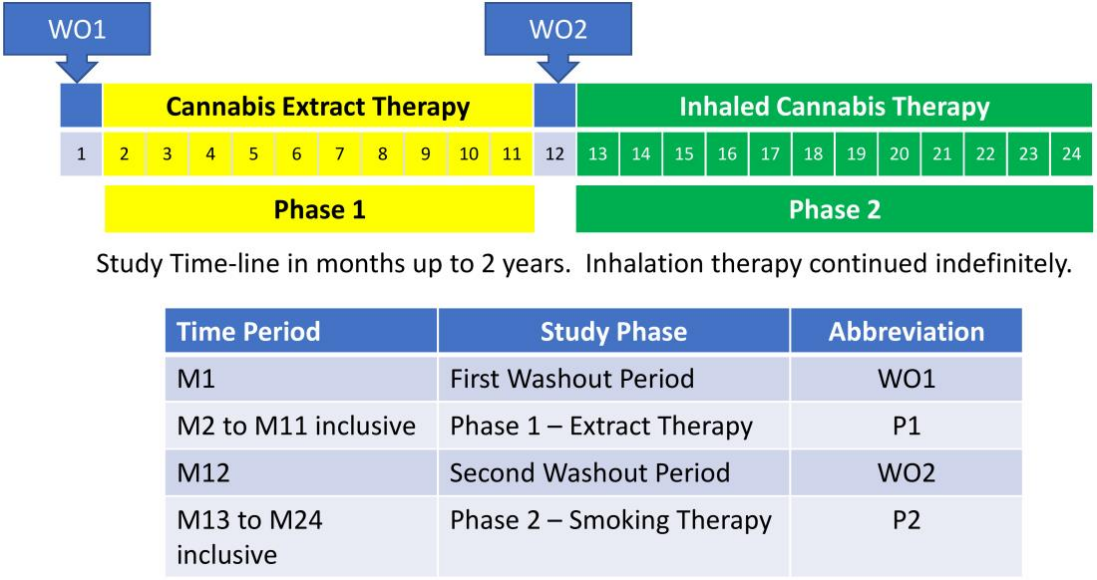
Study Timeline. A washout period of 1 month (WO1) was followed by 10 months of cannabis extract therapy, a second 1-month washout period (WO2), and a minimum of 12 months’ inhalation therapy, which was continued indefinitely, with follow-up every 6 months.

After study enrollment, all patients underwent an initial one-month washout period (WO1) to ensure they were in a cannabis-free symptom steady state. During WO1, analgesic medication regimens were continued as before, but all cannabis use was prohibited. After WO1, phase 1 (P1) of the study was initiated and sublingual cannabis extract was administered for 10 months using a personally adjusted slow titration protocol for each participant, based on clinical evaluations. At the end of 10 months, all participants underwent a second one-month washout period (WO2), after which phase 2 (P2) was initiated and patients were switched to smoked cannabis. This latter treatment continued indefinitely, with subsequent evaluations performed every six months (Figure 1).

### Analgesics Used During the Study

Study participants were allowed to use pain rescue medications as follows: tramadol (up to 300 mg per day), oxycodone (10 mg, three times a day [TID]), or acetaminophen (325 mg, TID).

### Medical Cannabis-based Products

Cannabis-based products were supplied by an IMOH-approved medical cannabis manufacturer; this ensured administration of products similar to those available in Israeli pharmacies serving medical cannabis patients for all approved indications. It should be noted that, in Israel, manufacturers are required to label THC, CBD, and cannabinol content and the dates of manufacture, packaging, and expiration. No other labeling regarding other phytoconstituents is currently required by law, despite their bioactive activity and effects.

The cannabis extract used for P1 was a supracritical CO_2_ extract of CBD-rich cultivar (CBD 16%: THC 0%), to which a supracritical CO_2_ extract from a THC-rich hybrid cultivar was added (THC 15%, CBD >0.5%), for a final CBD:THC ratio mix of 6:1. The initial concentration was 30 mg CBD and 5 mg THC per milliliter (20 drops per mL).

During P2, the initial inhaled cannabis dosage was 20 g dried inflorescences per month (~600 mg/day), as balanced mixed cultivars with a final concentration of 10%±4% THC:10%±4% CBD (equivalent to 36–84 mg of either THC or CBD). Two inflorescence brands were initially recommended to study participants: Argaman (Seach Medical Group, Givat Hen, Israel) or Paris (IM Cannabis, Tel Aviv, Israel).

At one month after inflorescence-use initiation, the concentration was modified, when clinically indicated, to 20%±4% THC:4%±4% CBD (corresponding to 96–144 mg THC:0–48 mg CBD, respectively, per day). Dose increase criterion was based on a Brief Pain Inventory (BPI) intensity score or interference score >6.

The recommended regimen ranged between 10 g and 20 g of Indica chemovar and 10 g of Sativa chemovar. The recommended Sativa brands for Sativa were either Tchelet brand (Seach Medical Group, Givat Hen, Israel) or Alaska brand (Tikun Olam, Tel Aviv, Israel). The recommended Indica chemovars were either Erez brand (Tikun Olam, Tel Aviv, Israel) or Roma brand (IM Cannabis, Tel Aviv, Israel(. These recommended brands were selected based on previous positive reviews by users of these chemovars.

### Dosage Regimen

#### Phase 1: Cannabis extract dosing

A dosing protocol for slow titration was implemented, and the daily cannabis extract dose was administered sublingually in equal parts, TID. The initial daily dose (dose level 1) was 5 mg CBD:0.833 mg THC. Every week, the total daily dose was increased, starting at 10 mg CBD:1.67 mg THC after one week, 20 mg CBD:3.4 mg THC after two weeks, and the maximal dose of 30 mg CBD:5 mg THC TID after three weeks. This last-mentioned dose was administered until the end of the 10-month cannabis extract treatment period.

If a participant experienced side effects or intolerance, the dose was reduced to the previously tolerated level. When possible, and based on the physician’s evaluation of the participant, another dose escalation was tried after three days.

#### Phase 2: Smoked cannabis dosing

Before beginning phase 2, patients were advised not to use cannabis with tobacco. The recommended dosage schedule was from TID up to four times a day. Participants were informed that Indica-based chemovars were preferred for use at night and hybrid/Sativa-weighted chemovars during the day.

The initial smoked cannabis regimen comprised dried inflorescences conforming to IMOH class 10% CBD:10% THC dosage. Participants were provided information regarding the recommended chemovars.

Therapy was initiated at 0.6 g per day per the IMOH guidelines, representing a theoretical daily dose of 100 mg CBD:100 mg THC. However, since only part of the smoked cannabis enters the lungs, the actual inhaled amount is less.^16^,^17^ Therefore, precisely determining the amount absorbed into the bloodstream is difficult.

An increased concentration was considered based on a pain questionnaire completed by each patient. After a minimum of 1 month, a dosage increase was considered for patients whose pain was unresponsive to inhaled therapy (up to 30 g of balanced inflorescences). According to clinical need, following three months of smoking therapy, the treatment concentration was changed to 20%±4% THC and 4%±4% CBD (30 g). At subsequent time-points, dosages were increased in 10-g increments every three months, up to a maximum dose of 60 g inflorescences per month (representing around 2 g per day). Dosage increase was considered only if clinically indicated according to BPI scores.

### Patient Assessment

Patients were assessed at study enrollment, at the end of WO1, and at 3, 6, 11 (before WO1), 12 (after WO1), 18, and 24 months thereafter. Participant assessment data included body weight, blood pressure, a general physical examination, and completion of the following questionnaires: (1) patient-reported outcome SF-12 version-1 questionnaire (SF-12v1), a health-related quality of life questionnaire that assessed health domains and evaluated both a physical component score (PCS) and the mental component score (MCS)^18^; (2) the Oswestry LBP Disability Questionnaire (also known as Oswestry Disability Index [ODI]), considered a gold-standard for measuring LBP functional outcomes, as it evaluates permanent functional disability on a scale from 1 (normal function) to 100 (full disability)^19^; and (3) the pain rating Visual Analog Scale (VAS),^20^ based on self-reported pain on a scale of 0 (no pain) to 10 (worst imaginable pain), by answering the question, “How do you score the highest pain intensity you have experienced during the last one week?” As mentioned above, the BPI was used to assess dose adequacy but not as a study endpoint.

During the study, analgesics consumption and changes in usage patterns were recorded and converted into morphine-equivalent units (MEU) per the Faculty of Pain Management of the Royal College of Anaesthetists guidelines.^15^

### Statistical Analysis

Statistical analysis was performed using Analyse-it for Excel 5.90 (2021) (Analyse-it Software, Ltd, Leeds, UK). Questionnaire scores were analyzed by analysis of variance (ANOVA) for repeated measures to determine any significant differences between data groups. The Tukey–Kramer all-pair comparison was used to ensure the significance of any particular-pair difference. Treatment of missing values was by the last observation carried forward (LOCF) method as described by Mavridis et al.,^21^ and was carried forward until the next available result or until the end of the study.

## RESULTS

### Patients and Patient Data

A total of 24 patients were enrolled in this study. No patients dropped out during washout-1 before commencing CB therapy. The average age was 46.1±21 years (range 18–91). The participant sex distribution was 17 males and 7 females; body mass index averaged 27.9±5.1.

Participants had an average of 2±1 concomitant chronic disorders as follows: diabetes (*n*=8), hypertension (*n*=9), hypercholesterolemia (*n*=6), moderate chronic obstructive pulmonary disease (*n*=2), ischemic heart disease (*n*=6), and peripheral vascular disease (*n*=1); one patient was a cancer survivor (in remission >2 years; no evidence of existing metastatic disease).

Advanced imaging studies confirmed possible pain-causing abnormalities in all participants, as follows: bulging discs (*n*=24), herniated discs (*n*= 16), sequestered discs (*n*=2), spinal stenosis (*n*=7), degenerative facet disease (*n*=5), grade 1 spondylolisthesis (*n*=2); no participants had evidence of a spinal neoplasm.

### Adherence to Cannabinoid-based Treatment

**Figure 2:**
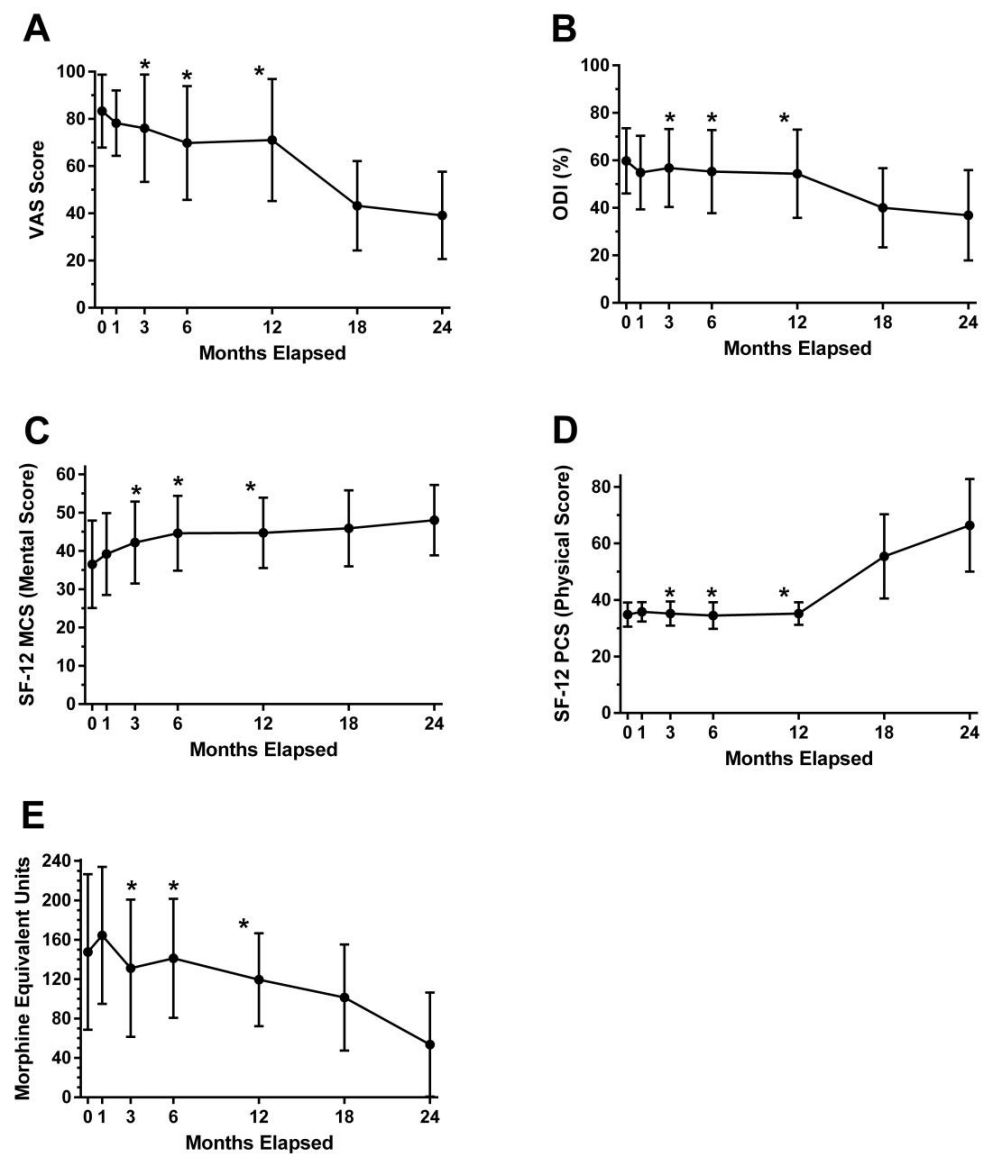
Summary of Participant Evaluations Throughout the Study Period (*n*=24*). **A:** Visual analogue scale (VAS). **B:** Oswestry Disability Index (ODI); **C:** SF-12 Mental Component Score (MCS) (version 1). **D:** SF-12 Physical Component Score (PCS). **E:** Morphine Equivalent Units (MEU) opiate usage. Results are expressed as mean±standard error (SE). Refer to Figure 1 for timing details. * Three patients stopped treatment during phase 1 only (sometime between months 2 and 11), but remained part of the evaluations throughout the study.

During phase 1 with extract treatment, 3 patients paused participation in extract therapy due to insufficient pain alleviation (Figures 1 and 2); however, they continued to be followed throughout. These 3 patients underwent observation until they stopped taking the extract; their last observation was carried forward to the end of the extract treatment phase (12 months from the enrollment to the study). Nonetheless, all 24 patients participated in WO2 and P2. Once smoked treatment P2 began, the originally planned follow-up evaluations and data collection were also resumed.

The VAS pain rating score decreased for all participants overall during the study, from 83.3± 15.4 at month 0 to 39.1±18.5 at 24 months (ANOVA *P*<0.001). During the extract therapy phase, this decrease was not significant and averaged 12.3% (standard error [SE] 5.8, confidence interval [CI] 95% −5.3–29.8). The change in VAS was significant at 12–24 months and 12–18 months (Figure 2A; supplemental Table 1).

A similar trend was observed for the ODI, which decreased from 59.8±13.8 at month 0 (prior to WO1) to 36.9±19 at 24 months (ANOVA *P*<0.001). Although the ODI decrease during P1 was not significant and averaged 5.4% (SE 5.8, CI 95% −9.1 to 20.0), the decrease became significant for months 12–24 and 12–18 (Figure 2B; supplemental Table 2).

**Table 2:** Cannabis Dosage During the Study and Adverse Effects.

Time-point	g/Month	Pts (*n*)	%THC:%CBD	Males:Females	Adverse Events
M0 (WO1)	--	24	--	17:7	
P1 M1	20	24	05THC:30CBD	17:7	Nausea, drowsiness, dizziness, fatigue
P1 M3	30	23	05THC:30CBD	16:7	Nausea, drowsiness, dizziness, fatigue
P1 M6	30	21	05THC:30CBD	16:5	Nausea, drowsiness, dizziness, fatigue
P2 M11 (WO2)	--	21	--	17:7	None
P2 M12	20	24	10THC:10CBD	17:7	Drowsiness, sore throat
P2 M18	30	20	20THC:04CBD	15:5	None
	40	2	20THC:04CBD	2 males	None
	20	2	10THC:10CBD	2 females	None
P2 M24	30	10	20THC:04CBD	5:5	None
	40	7	20THC:04CBD	7 males	None
	50	3	20THC:04CBD	3 males	None
	60	2	20THC:04CBD	2 males	None
	20	2	10THC:10CBD	2 females	None

M, month; M0, month 0 (before study start); P1, phase 1 of study (extract therapy); P2, phase 2 of study (smoked); Pts, patients.

The MCS improvement was significant over the study’s duration, with an average of 36.5±11.4 at month 0 and improving to 48±9.2 at 24 months (ANOVA *P*<0.002). There was a significant difference between months 0, 18, and 24. Additionally, a trend for significance was found for the difference between 0–6 and 0–12 months (*P*>0.09) (Figure 2C). The PCS difference was significant at several time-points (Figure 2D and corresponding supplemental Tables 3 and 4). Again, no significant difference was noted from 0 to 12 months (P1).

### Cannabis Dosage

The 21 patients who completed P1 reached the maximal planned extract dosage (30 mg CBD:5 mg THC) (Table 2). This was maintained throughout the study without complications. At 24 months, the cannabis concentration was 20% THC for 22 patients (Table 2), while two patients (both females) preferred to continue with 10% THC concentration. No patients dropped out during P2.

During P1 there was a minimal (non-significant) decline in analgesic usage patterns. However, during P2, a significant decline of 65.8±18.1 MEU (95% CI 11.7–117.1) was observed (*P*<0.007) (Figure 2E).

### Adverse Events

While all 24 patients reported experiencing “red eyes,” no serious adverse events were recorded. No patient underwent surgery or had required spinal injections during the study period. Reported minor adverse events were: nausea, dizziness, and fatigue during the extract phase; sore throat during the inhalation phase; and drowsiness during both phases (Table 2).

## DISCUSSION

The results of this study suggest that CB therapy is an efficacious treatment for LBP. First et al. reviewed 124 research papers and noted a sparsity of information regarding the effect of cannabis therapy on LBP symptomatology.^22^ Only a few studies have investigated the effects of medical CB therapy directly on chronic LBP (including our previous studies).^12^,^23^

Our results also indicated that THC-rich smoked-cannabis inflorescence was more effective than CBD-rich cannabis-extracts for inducing symptom relief in LBP. However, due to the study design, it was impossible to rule out the possible clinical benefit of the extracts, which may have required a longer treatment duration. In addition, since the P2 smoked-cannabis therapy followed P1 extract therapy, it is possible that the longer duration of P2 treatment led to the eventual improvement in LBP symptoms.

The dried inflorescences dispensed to patients in our study had a higher THC concentration and lower CBD concentration; hence, our results suggest that THC has a positive effect in alleviating pain when consumed in accordance with the study protocol. Furthermore, the results of this study suggest that a high THC:CBD ratio is more advantageous for a therapeutic effect in LBP. Interestingly, a meta-analysis of 33 orthopedic studies suggested that results appeared to be efficacious in non-controlled studies, while appearing mixed in controlled randomized studies^24^; furthermore, like the results of this current study, the authors found that studies using higher doses of cannabis tended to conclude that its use was effective, notwithstanding the potential for an increase in harmful effects with higher doses.^24^

A retrospective study of cannabis extracts demonstrated anecdotal evidence of decreased pain levels and improved sleep using THC-rich cannabis extract preparations (up to 82% THC)^25^; the authors concluded that cannabis galenical preparations may be both safe and therapeutically effective for symptomatic treatment of some chronic diseases.^26^ Such concentrated oils are currently not allowed in Israel; however, the observed improved results may be attributed to the high THC concentration and quantity that was administered (0.5–1.0 mL per day of 82% THC oil, i.e. up to 410–820 mg THC per day). However, such a high THC dosage carries the risk of a possible increase of adverse events and their severity. Our current study had a very good safety profile with no serious adverse events, and only minor common adverse events were reported.

With regard to pain, one study has suggested further investigation of cannabigerol (CBG)-predominant CB treatments (>50% CBG), also unavailable in Israel, since 40.9% (*n*=127) of its patients who reported using CBG-predominant CB products to treat chronic pain had significantly less withdrawal symptoms compared to non-CBG-predominant products.^27^

This current study included 7 (29%) females and 17 males (71%). The most commonly reported adverse events during P1 were nausea, sore throat, drowsiness, dizziness, and fatigue, all of which were transient and disappeared after dose tolerance was achieved. Most of these adverse effects were noted in female patients. Interestingly, females have been reported to have more adverse effects than males, despite presenting with pain intensities similar to those of males.^28^ Another study reported that 1,675 (34.2%) of 4,891 respondents (no sex distribution) had at least one side effect (most commonly reported were dizziness, dry mouth, increased appetite, and sleepiness), with no serious adverse effects.^13^

Cannabidiol was also evaluated with regard to LBP. Two recent reviews noted that patients had reported a beneficial effect on chronic pain after CBD treatments; both reviews also noted that studies of CBD-rich treatments for pain were both scarce and limited, since dosing, dosing frequency, or dose combinations were not fully explored.^4^,^29^ Nonetheless, the National Institute for Clinical Excellence has made a research recommendation for CBD in adults suffering from fibromyalgia or treatment-resistant neuropathic pain.^30^ Interestingly, a recent survey found that 72% of 878 fibromyalgia patients who received CBD treatments instead of standard pharmaceutical therapy noted an improvement in their condition,^11^ similarly to our previous findings in a study involving 31 fibromyalgia patients suffering from LBP, which also showed an advantage of CB therapy when compared to standard analgesic therapy.^23^

The results of our study showed a significant improvement in LBP, as demonstrated by VAS, ODI, MCS, and PCS scores at 24 months. When comparing our results to those of other studies, it is important to note that some of the results were non-significant at the 12-month time-point. Nevertheless, another study with 4,166 participants receiving various cannabis treatments for different indications demonstrated that the pain intensity at six months was improved for 74.7% of their cohort and did not change for 17.8%. Moreover, of their 1,580 patients treated for CNNCP, 85.9% experienced a ≥30% improvement on the VAS pain scale, and 59.3% a >50% improvement.^13^

The high variability existing between and among cannabis strains and chemovars complicates defining the “ideal” strain in a specific clinical situation.^6^,^31^ Aviram et al. illustrate the complex multiplicity of treatment that can be found in different studies.^28^ From a cohort of 429, they noted that smoking was preferred by 76% of the patients, vaporizing by 20%, and 4% preferred alternating both routes of administration. Moreover, with regard to sex and CNNCP, the study noted that a total of 41 cultivars and 222 cultivar combinations were used, of which 50% were unique to males, 32% unique to females, and 18% were used by both sexes. They also noted that females had consumed different combinations than the males, with higher monthly doses of CBD and cannabichromene (CBC), and significantly lower doses of 373-15c-phytocannabinoid and the terpenoid linalool, while males had consumed higher monthly doses of cannabinol (CBN) and the terpenoid beta-myrcene and lower monthly dose of 331-18b-phytocannabinoid.^28^

A recent six-month observational study (*n*= 4,364) in which patients had limited CB product options from which to choose also demonstrates the complex multiplicity of treatment options.^13^ The authors noted a significantly different treatment success rate when analyzing 1,500 patients who used only one chemovar. The varied treatment success rate was attributed to the patients’ different medical conditions.^13^ The same study also reported that 2,551 (55.7%) patients used a specific 18% THC Indica chemovar, of which 1,306 patients received inhalation therapy (average: 0.3 g [54 mg THC] inflorescence, 3.4 times a day), and 935 patients were treated with the same chemovar extract (average dose of 5.7 mg TCH per administration, 2.4 times a day).^13^

In our study, six months into P1, 21 participants had achieved the maximal dosage of 1 mL extract (30 mg CBD:5 mg THC), which had been titered up from 10 mg THC:10 mg CBD extracts; 3 participants (2 females, 1 male) had dropped out due to insufficient pain relief (Figure 2). During P1, 2 female study participants preferred the 10% THC:10% CBD inflorescences over a higher THC concentration. Interestingly, a study (*n*=151) evaluating the benefits of 10 mg THC:10 mg CBD cannabis extract on chronic refractory pain over 3 months reported improved pain impact scores in 47.9% of participants, unchanged scores in 28.8%, and worse scores in 23.3%; pain intensity had improved for 32.9%, remained unchanged for 45.2%, and worsened for 21.9% of the participants. The authors suggested that the reduced pain intensity influenced the patients’ quality of life, which was not reflected in the pain intensity measurements.^32^ Another observational study (*n*=3,544) reported a reduction of pain medications at 6 months, including reduction in opioid use by 52.5%, in other analgesics by 39.2%, in anti-psychotics by 36.9%, in anti-epileptics by 35.7%, and in hypnotics and sedatives by 35.3%.^13^

The monthly CB dosage distribution of all patients in the current study (Table 2) was relatively similar to the national data reported by the Israeli Medical Cannabis Unit in their January 2022 report of the general patient population in Israel (including CNNCP patients): 21–30 gram licenses were highest, representing 26.51% of total licenses issued, compared to 41.6% in our study; 31–40 gram licenses represented 23.82% compared to 29.16% in our study; 41–50 gram licenses were 17.04% compared to 12.5% in our study; and 51–60 gram licenses were issued to 8.13% compared to 8% in our study.^2^,^3^

## LIMITATIONS

In Israel there is no actual control over CB product purchases, and patients may choose from a variety of approved CB products in accordance with their cannabis prescription, which can be filled by authorized pharmacies. A standard prescription specifies the product types, grams per month, and THC:CBD ratio permitted, but not the other bioactive phytoconstituents of cannabis such as terpenes or flavonoids, which may influence treatment outcomes. Patients in Israel are not required to report which product they purchase, and CB labeling does not provide data regarding phytoconstituents other than THC, CBD, and cannabinol. Similarly, our study did not analyze CB products for chemical or toxicological phytoconstituents; hence, the chemovar characteristics that may have been beneficial to participants could not be determined. To minimize potential participant dissatisfaction with specific chemovars, the authors recommended specific ones based on their previous experience with those chemovars in LBP patients. While Israeli patients are not bound by this recommendation, they do tend to adhere to the recommended chemovar when available at pharmacies. A patient’s freedom to choose any chemovar is a disadvantage of the current study; however, this study represents real-world experience as distinguished from participants being supplied with a single chemovar. While this created some undesirable treatment variability, it increased the likelihood of participant compliance.

Another study limitation was the sample size. There are few studies that directly assess the effectiveness of CB treatments for LBP, and even fewer studies of two years’ duration and above. This study aimed to contribute to the growing knowledge in the field, and to support further studies involving larger numbers of patients for a longer duration, and with different cohorts to further elucidate knowledge regarding CB treatment modalities. With regard to sex distribution, although the male:female ratio in this study favored male patients, we do call for sex-balanced studies for the benefit of the general population.

## SUMMARY

The current study is the first, to our knowledge, to indicate that THC-rich smoked therapy is more advantageous in ameliorating LBP, than low THC CBD-rich sublingual extracts. Despite the small number of patients, our data indicate that THC-rich smoked therapy is helpful in mitigating LBP. The relative strength of this study was its medium duration (up to 2 years), patient follow-ups, and multidimensional assessments that included physical and mental function, and pain and disability assessment. Further studies should be performed to assess the possible effect of THC-rich sublingual extracts compared with CBD-rich sublingual extracts for LBP treatment.

## Supplementary Information



## Abbreviations

ANOVA: analysis of variance
CB: cannabinoid-based
CBD: cannabidiol
CBG: cannabigerol
CNNCP: chronic neuropathic non-cancer pain
CT: computed tomography
IMOH: Israeli Ministry of Health
LBP: low back pain
MCS: mental component score
MRI: magnetic resonance imaging
ODI: Oswestry Disability Index
PCS: physical component score
SF-12: SF-12 patient reported outcome questionnaire
THC: Δ^9^-tetrahydrocannabinol
TID: three times a day
VAS: visual analogue scale.
