# Supplementary material for: Comparing Sublingual and Inhaled Cannabis Therapies for Low Back Pain: An Observational Open-Label Study
Source: Rambam Maimonides Med J. 2022 Oct 27;13(4):e0026. doi: 10.5041/RMMJ.10485 (PMC9622393; doi:10.5041/RMMJ.10485)
Supplement: Supplementary file 1 [file rmmj-13-3-e0026-am.pdf]

SUPPLEMENTARY MATERIAL

This appendix has been provided by the authors for the benefit of readers

Supplement to: Comparing Sublingual and Inhaled Cannabis Therapies for Low Back Pain: An Observational Open-Label Study

Robinson D, Ritter S, Yassin M. Comparing Sublingual and Inhaled Cannabis Therapies for Low Back Pain: An Observational Open-Label Study. Rambam Maimonides Med J 2022;13(4):e0026. doi:10.5041/RMMJ.10485

Supplemental Tables 1–4 appears on the following pages. For clarity, the reader should refer to Figure 1 of the main manuscript for a detailed explanation of the study timeline.

Supplemental Table 1. Visual Analogue Scale (VAS) Results per Time-point; Tukey-Kramer All Pairs Comparisons.

Contrast	Mean Difference	Simultaneous 95% CI	Standard Error	*P* Value
0-24 months	40.0	26.6-61.7	5.87	<0.0001 [1]
0-18 months	39.0	22.5-57.6	5.87	<0.0001 [1]
1-24 months	36.9	21.5-56.6	5.87	<0.0001 [1]
3-24 months	35.0	19.3-54.4	5.87	<0.0001 [1]
1-18 months	32.8	17.4-52.5	5.87	<0.0001 [1]
3-18 months	31.9	15.3-50.3	5.87	<0.0001 [1]
12-24 months	30.7	14.3-49.4	5.87	<0.0001 [1]
6-24 months	27.8	13.1-48.2	5.87	<0.0001 [1]
12-18 months	26.6	10.3-45.3	5.87	<0.0001 [1]
6-18 months	13.5	9.1-44.1	5.87	0.0002 [1]
0-6 months	12.3	-4.1-31.0	5.87	0.2542 [2]
0-12 months	8.4	-5.3-29.8	5.87	0.3657 [2]
1-6 months	7.3	-9.2-25.9	5.87	0.7868 [2]
0-3 months	7.2	-10.3-24.8	5.87	0.8795 [2]
1-12 month	6.2	-10.4-24.7	5.87	0.8852 [2]
3-6 months	5.1	-11.3-23.7	5.87	0.9394 [2]
0-1 month	5.0	-12.4-22.6	5.87	0.9770 [2]
3-12 months	4.1	-12.5-22.5	5.87	0.9789 [2]
18-24 months	2.2	-13.4-21.6	5.87	0.9927 [2]
1-3 months	1.2	-15.4-19.7	5.87	0.9998 [2]
12-6 months		-16.3-18.7	5.87	1.0000 [2]

H0: $\theta = 0$, the difference between the means of the populations is equal to 0. H1: $\theta \neq 0$, the difference between the means of the populations is not equal to 0.

[1] Reject the null hypothesis in favor of the alternative hypothesis at the 5% significance level.

[2] Do not reject the null hypothesis at the 5% significance level.

A significant difference in VAS was found from baseline to 18 months and 24 months but not to 12 months. A significant difference was found comparing 3 and 6 months with 18 and 24 months. This indicates the superiority of the inhalation cannabis therapy as compared with cannabis extract therapy.

Supplemental Table 2. Oswestry Disability Index (ODI) Results per Time-point; Tukey-Kramer All Pairs Comparisons.

Contrast	Mean Difference	Simultaneous 95% CI	Standard Error	*P* Value
0-24 months	23	23-8.4	4.87	0.0001 [1]
3-24 months	19.9	19.9-5.3	4.87	0.0014 [1]
0-18 months	19.8	19.8-5.2	4.87	0.0015 [1]
6-24 months	18.4	18.4-3.8	4.87	0.0042 [1]
1-24 months	18	18-3.5	4.87	0.0054 [1]
12-24 months	17.5	17.5-3	4.87	0.0076 [1]
3-18 months	16.7	16.7-2.2	4.87	0.0134 [1]
6-18 months	15.2	15.2-0.7	4.87	0.0342 [1]
1-18 months	14.9	14.9-0.3	4.87	0.0416 [1]
12-18 months	14.4	14.4-(-0.2)	4.87	0.0553 [2]
0-12 months	5.4	5.4-(-9.1)	4.87	0.9239 [2]
0-1 month	4.9	4.9-(-9.6)	4.87	0.9514 [2]
0-6 months	4.6	4.6-(-10)	4.87	0.9654 [2]
18-24 months	3.2	3.2-(-11.4)	4.87	0.9950 [2]
0-3 months	3.1	3.1-(-11.5)	4.87	0.9956 [2]
3-12 months	2.3	2.3-(-12.2)	4.87	0.9991 [2]
1-3 months	1.8	1.8-(-12.7)	4.87	0.9998 [2]
3-6 months	1.5	1.5-(-13.1)	4.87	0.9999 [2]
6-12 months	0.8	0.8-(-13.7)	4.87	1.0000 [2]
1-12 months	0.5	0.5-(-14.1)	4.87	1.0000 [2]
1-6 months	0.3	0.3-(-14.2)	4.87	1.0000 [2]

H0: $\theta = 0$, the difference between the means of the populations is equal to 0. H1: $\theta \neq 0$, the difference between the means of the populations is not equal to 0.

[1] Reject the null hypothesis in favor of the alternative hypothesis at the 5% significance level.

[2] Do not reject the null hypothesis at the 5% significance level.

The ODI improved from baseline to 18 and 24 months. The improvement was mostly during the inhalation therapy stage of the study (P2).

Supplemental Table 3. Mental Component Score (MCS) Results per Time-point; MCS-SF-12 Tukey-Kramer All Pairs Comparisons.

Contrast	Mean Difference	Simultaneous 95% CI	Standard Error	*P* Value
0-24 month	11.5	2.7-20.3	2.93	0.0024 [1]
0-18 month	9.4	0.7-18.2	2.93	0.0261 [1]
1-24 month	8.8	0-17.5	2.93	0.0482 [1]
0-12 month	8.2	-0.6-16.9	2.93	0.0848 [2]
0-6 month	8.1	-0.6-16.9	2.93	0.0879 [2]
1-18 month	6.7	-2-15.5	2.93	0.2560 [2]
3-24 months	5.8	-2.9-14.6	2.93	0.4251 [2]
0-3 month	5.7	-3.1-14.4	2.93	0.4617 [2]
1-12 month	5.5	-3.3-14.2	2.93	0.5085 [2]
1-6 month	5.4	-3.3-14.2	2.93	0.5180 [2]
3-18 months	3.8	-5-12.5	2.93	0.8605 [2]
6-24 months	3.4	-5.4-12.1	2.93	0.9109 [2]
12-24 months	3.3	-5.4-12.1	2.93	0.9157 [2]
1-3 months	3	-5.8-11.7	2.93	0.9513 [2]
0-1 month	2.7	-6-11.5	2.93	0.9682 [2]
3-12 months	2.5	-6.3-11.3	2.93	0.9787 [2]
3-6 months	2.5	-6.3-11.2	2.93	0.9805 [2]
18-24 months	2.1	-6.7-10.9	2.93	0.9918 [2]
6-18 months	1.3	-7.5-10.0	2.93	0.9994 [2]
12-18 months	1.3	-7.5-10.0	2.93	0.9995 [2]
6-12 months	0	-8.7-8.8	2.93	1.0000 [2]

H0: $\theta = 0$, the difference between the means of the populations is equal to 0. H1: $\theta \neq 0$, the difference between the means of the populations is not equal to 0.

[1] Reject the null hypothesis in favor of the alternative hypothesis at the 5% significance level.

[2] Do not reject the null hypothesis at the 5% significance level.

The mental subscale of the SF-12 (MCS) significantly improved from baseline and 1 month, to 18 and 24 months. The improvement was not significant from 0 to 6 months and from baseline to 12 months (though a trend was indicated by a *P* value smaller than 0.1).

Supplemental Table 4. SF-12 Physical Component Score (PCS) Results per Time-point; Tukey-Kramer All Pairs Comparisons.

Contrast	Mean Difference	Simultaneous 95% CI	Standard Error	*P* Value
6-12 months	32	24.1–39.8	2.62	<0.0001 [1]
0-24 months	31.6	23.7-39.4	2.62	<0.0001 [1]
12-24 months	31.3	23.4-39.1	2.62	<0.0001 [1]
3-24 months	31.2	23.4–39.0	2.62	<0.0001 [1]
1-24 months	30.7	22.8–38.5	2.62	<0.0001 [1]
6-18 months	21	13.1–28.8	2.62	<0.0001 [1]
0-18 months	20.6	12.7-28.4	2.62	<0.0001 [1]
12-18 months	20.3	12.4–28.1	2.62	<0.0001 [1]
3-18 months	20.2	12.4–28.0	2.62	<0.0001 [1]
1-18 months	19.7	11.8–27.5	2.62	<0.0001 [1]
18-24 months	11	3.2–18.8	2.62	0.0009 [1]
1–6 months	1.3	-6.5–9.1	2.62	0.9989 [2]
0-1 month	0.9	-6.9–8.8	2.62	0.9999 [2]
3–6 months	0.8	-7.1–8.6	2.62	1.0000 [2]
12–6 months	0.7	-7.1–8.5	2.62	1.0000 [2]
1–12 months	0.6	-7.3–8.4	2.62	1.0000 [2]
1–3 months	0.5	-7.3–8.4	2.62	1.0000 [2]
0-3 months	0.4	-7.5–8.2	2.62	1.0000 [2]
0–6 months	0.4	-7.5–8.2	2.62	1.0000 [2]
0-12 months	0.3	-7.5–8.2	2.62	1.0000 [2]
3-12 months	0	-7.8–7.0	2.62	1.0000 [2]

H0: $\theta = 0$, the difference between the means of the populations is equal to 0. H1: $\theta \neq 0$, the difference between the means of the populations is not equal to 0.

[1] Reject the null hypothesis in favor of the alternative hypothesis at the 5% significance level.

[2] Do not reject the null hypothesis at the 5% significance level.

The physical subscale of the SF-12 (PCS) significantly improved compared to the baseline, 3 months, 6 months to 18- and 24-months therapy. This indicates the possible superiority of the inhalation therapy compared with extract therapy. There was a significant improvement from 18 to 24 months, possibly indicating that the improvement in physical function had not yet plateaued; it is possible that longer follow-up is needed to achieve plateau.
